# Transcription factorIRX5 promotes hepatocellular carcinoma proliferation and inhibits apoptosis by regulating the p53 signalling pathway

**DOI:** 10.1002/cbf.3517

**Published:** 2020-03-09

**Authors:** Liying Zhu, Longguang Dai, Nenghong Yang, Mi Liu, Shuang Ma, Chengcheng Li, Jie Shen, Tao Lin, Dan Wang, Wei Pan, Xing Li

**Affiliations:** ^1^ Affiliated Hospital of Guizhou Medical University Guizhou Medical University Guiyang China; ^2^ Guizhou university of traditional Chinese medicine Guiyang China; ^3^ Clinical Laboratory The Tumor Hospital of Guizhou Province Guiyang China; ^4^ Clinical Laboratory Guizhou Provincial People^,^s Hospital Guiyang China; ^5^ Department of Clinical Laboratory The People's Hospital of Rongchang District Chongqing China

**Keywords:** apoptosis, hepatocellular carcinoma, IRX5, p53 signalling pathway, proliferation

## Abstract

Hepatocellular carcinoma (HCC) is the fifth most common cancer worldwide and the third most frequent cause of cancer‐related death. The IRX5 transcription factor plays a different role in multiple cancers and contributes to the development of many tumours. However, little is known about the molecular mechanisms of IRX5 in HCC. In this study, we found that IRX5 was abnormally upregulated in HCC tissues compared with adjacent normal tissues. IRX5 promoted HCC cell proliferation and upregulated the expression of cyclin D1 and knockdown of IRX5 suppressed tumorigenicity *in vivo*. Furthermore, knockdown of IRX5 increased p53 and Bax expression and decreased Bcl‐2 expression. Thus, IRX5 suppressed apoptosis in HCC cells by inhibiting the p53 signalling pathway, indicating its role as a treatment target for HCC.

**Significance of the study:**

Our study demonstrated that IRX5 was abnormally upregulated in HCC tissues compared with adjacent normal tissues. IRX5 promoted HCC cell proliferation and upregulated the expression of cyclin D1, and knockdown of IRX5 suppressed tumorigenicity *in vivo*. Furthermore, knockdown of IRX5 increased p53 and Bax expression and decreased Bcl‐2 expression. IRX5 suppressed apoptosis in HCC cells by inhibiting the p53 signalling pathway, indicating its role as a treatment target for HCC.

## 1 INTRODUCTION

Liver cancer was the sixth most commonly diagnosed cancer and the fourth leading cause of cancer‐related death worldwide in 2018,^1^ with approximately 841 000 new cases and 782 000 deaths annually.^2^ Hepatocellular carcinoma (HCC) accounts for 75‐85% of primary liver cancers and is one of the most common malignant tumours in the world.^3^ Surgery is the most effective therapy for HCC at present.^4^ The vast majority of HCC patients present with advanced‐stage disease that ultimately leads to poor prognosis^5^ and a 5‐year survival rate of less than 20%^4^, ^6^; the recurrence rate is as high as 80%.^7^ The mechanism of HCC development has not been clarified, particularly the mechanism underlying the regulation of the proliferation and apoptosis of HCC.

Iroquois homeobox (Irx) genes play pivotal roles in normal embryonic cell patterning and the development of malignancies.^8^ The Irx family is composed of six genes in humans and mice, including two clusters, IrxA (Irx1, Irx2 and Irx4) located on chromosome 5 and IrxB (Irx3, Irx5 and Irx6) located on chromosome 16.^9^ IRX5 encodes a transcription factor and is a highly conserved member of the Iroquois homeobox gene family.^8^ IRX5 plays a different role in multiple cancers, contributing to the development of many tumours by acting as an important transcription factor regulating key regulatory genes that control cell growth, invasion, migration and apoptosis. Recent data suggest that IRX5 is a transcription factor that remarkably promotes tongue squamous cell carcinoma tumour growth by targeting the osteopontin (OPN) promoter and activating the NF‐κB pathway.^8^ Our previous study showed that CRNDE acted as a tumour oncogene by promoting the oncogenic properties of human HCC and revealed a novel CRNDE‐miR‐136‐5p‐IRX5 regulatory network in HCC.^3^

CyclinD1 regulates cell cycle progression, forms complexes with cyclin‐dependent kinase 4 or 6 in the cytoplasm^10^, ^11^ and promotes progression from G1 to S‐phase.^12^ Previous findings have indicated that cyclin D1 is a downstream target of IRX5.^9^ P53 is a tumour suppressor gene,^13^ and the p53 protein is a transcription factor that is involved in cell cycle arrest, DNA repair and apoptosis.^14^ In the p53 signalling pathway, p53 positively regulates the proapoptotic proteins Bax and p53 negatively regulates the transcription of Bcl‐2.^15^

In this study, we explored the role ofIRX5 in HCC proliferation and apoptosis. Next, we will discuss the properties of IRX5 in the p53 signalling pathway.

## 2 MATERIALS AND METHODS

### 2.1 HCC cell lines

HepG2, SMMC7721, SK‐hep1, Huh7, human immortalized normal human liver cell line (L02) and the embryonic kidney cell line 293 T were obtained from the Chinese Academy of Sciences Cell Bank. They were cultured in Dulbecco's modified Eagle's medium (DMEM) of high glucose with 10% foetal bovine serum (FBS, BI, ISR). All cells were incubated at 37 °C in a humidified incubator with 5% CO_2_.

### 2.2 Human tissues

Ten pairs of primary HCC and adjacent non‐tumour tissues were obtained from surgical resections of HCC in the Affiliated Hospital of Guizhou Medical University between January 2015 and January 2016. Fresh tissue samples were collected and processed within 10 minutes. Each sample was snap‐frozen in liquid nitrogen and then stored at −196 °C. The data do not contain any information that could identify the patients. All patients provided written informed consent, and ethical consent was granted from the Committees for Ethical Review of Research involving the Affiliated Hospital of Guizhou Medical University (Guizhou, China).

### 2.3 Plasmid construction

PcDNA3.1‐IRX5 was purchased from GenePharma (Shanghai, China). The short‐hairpin RNA targeting human IRX5 was ligated into the pGreenPuro shRNA vector (SBI, Palo Alto, CA) according to manufacturer's protocol. The constructed sequence was further confirmed by sequencing. Transfections were performed with a Lipofectamine 2000 kit (Invitrogen, Carlsbad, CA), according to manufacturer's instructions. Cells were harvested 48‐72 hours after transfection.

### 2.4 Cell proliferation assays

Cells (2000 cells/well) were seeded into 96 well plates after 24 hours of transfection and measured at different time points (0, 24, 48 and 72 hours) using the MTS kit (CellTiter 96 Aqueous One Solution Cell Proliferation Assay, Promega), following the manufacturer's protocol. The absorbance at 490 nm was measured with a spectrophotometer. All experiments were performed in triplicate.

### 2.5 Reverse transcription and quantitative real‐time polymerase chain reaction

We extracted total RNA from HCC cells using TRIzol reagent (Life Technologies Corporation, Carlsbad, CA, USA) and determined RNA concentration and quality by the 260/280 nm ratio using a Nanodrop Spectrophotometer (ND‐2000, Thermo Fisher Scientific, Waltham, MA, USA). Next, RNA was reverse transcribed into cDNA and used for quantitative real‐time polymerase chain reaction (qRT‐PCR). We used glyceraldehyde 3‐phosphate dehydrogenase (GAPDH) as an internal control and expressed all of the results as the mean ± standard deviation (s.d.) of at least three independent experiments. Comparative quantification was determined using the 2^−ΔΔCt^ method. The primer sequences were as follows: GAPDH‐Forward: GTCTCCTCTGAC TTCAACA and GAPDH‐Reverse: GTGAGGGTCTCTCTCTTCCT; IRX5‐Forward: ACCCAGCGTACCGGAAGAA and IRX5‐Reverse:CGGCGTCCAC GTCATTTTAT.

### 2.6 Immunohistochemical staining

All tissues were fixed with 10% paraformaldehyde and embedded in paraffin wax. Paraffin sections were placed in incubators kept at 55 °C for 4 hours. The sections were immersed in two consecutive washes of xylol for 20 minutes to remove paraffin. Sections were then hydrated with different concentrations of ethanol including 100%, 95%, 85%, 70% and deionized water. Endogenous peroxidase was quenched with 3% H_2_O_2_ in methanol for 10 minutes and washed for 10 minutes in PBS. The sections were immersed in citrate buffer solution (0.01 mol l^−1^, pH 6.0) and heated to retrieve antigen. Then, 0.5%Triton‐x‐100 was incubated 30 minutes after washing with PBS. The tissues were blocked in 10% goat serum for 1 hour before the addition of the mouse monoclonal antibodies against IRX5 (diluted 1:150, Atlas，USA) and Ki‐67 (1:500, Abcam, Cambridge, UK) at 4 °C overnight. The sections were incubated with HRP‐conjugated goat anti‐mouse secondary antibody (diluted 1:1000, Bioword, USA) for 30 minutes and then with 3,3‐diaminobenzidine (DAB)/H_2_O_2_ for 5 minutes. Slides were imaged under a light microscope (Olympus, Japan) at ×400 magnification.

### 2.7 Western blot analysis

We extracted total proteins from the HCC cells using RIPA buffer and protease inhibitors (Solarbio, Beijing, China) according to manufacturer's protocol, and the protein concentrations were determined using a BCA assay kit (Solarbio, Beijing, China); protein were subjected to sodium dodecyl sulphate polyacrylamide gel electrophoresis and electrophoretically transferred to polyvinylidene fluoride membranes (Merck Millipore, MA, USA). Membranes were incubated in 5% nonfat milk dissolved in Tris‐buffered saline (TBS) containing 0.1% Tween‐20 for 2 hours at room temperature and then incubated with primary antibodies as follows: IRX5 (diluted 1:500, Abcam, Cambridge, MA, USA), GAPDH (diluted 1:2000, ProteinTech, Wuhan, China), Bcl‐2 (diluted 1:500, Affinity Biosciences, Changzhou, China), Bax (diluted1:2000,ProteinTech), cyclinD1 (diluted 1:500, Bioworld Technology, Nanjing, China) and p53 (diluted1:2000, ProteinTech).Then, the membranes were incubated with secondary antibodies (diluted 1:5000, Affinity Biosciences, Changzhou, China) at room temperature for 2 hours. Immunoblots were visualised by enhanced chemiluminescence (ECL kit; Advansta, Menlo Park, CA, USA) and scanned using an ECL chemiluminescence detection system (Pierce Biotechnology, Waltham, MA, USA). All experiments were performed in triplicate.

### 2.8 Cell cycle and cell apoptosis assays

Cell cycle and cell apoptosis assays were assays by using flow cytometry assays (BD, Franklin Lakes, NJ). The cells were harvested and then washed twice with PBS and resuspended in 100 μL of binding buffer. The cells were fixed in 70% ice‐cold ethanol and after holding overnight at 4 °C, the cells were supplemented with RNaseA (Keygen Biotech) and propidium iodide for 37 °C for 30 minutes. The DNA content of labelled cells was detected using FACS cytometry (BD Biosciences Inc., Franklin Lakes, NJ, USA). Cell apoptosis was assessed by using a flow cytometry assay (BD, Franklin Lakes, NJ). Each experiment was performed in triplicate.

### 2.9 Construction of stable cell lines

The short‐hairpin RNA targeting human IRX5 was ligated into the pGreenPuro™ shRNA vector (System Biosciences, Palo Alto, CA, USA) according to the manufacturer's protocol. Transfected SMMC7721 cells were selected with puromycin (2 μg ml^−1^) for 4 weeks. Selected cells were further subcloned for uniform stable cell lines. The stably interfering cell lines were identified using western blot.

### 2.10 Animal studies

Male BALB/c nude mice (4‐6 weeks old) were purchased from the Animal Care Committee of Chongqing Medical College. Ten mice were randomly allocated into two groups. A total of 1 × 10^7^ SMMC7721 cells stably transfected with IRX5‐interference (sh‐IRX5) and negative control shRNA (sh‐NC) were subcutaneously injected into the dorsal flanks of mice. After 28 days, the mice were sacrificed, and tumours were removed and photographed. The expression of IRX5 and Ki‐67 in Xenograft tumours was detected by immunohistochemistry.

### 2.11 Statistical analysis

SPSS 17.0 software (SPSS Inc., Chicago, IL, USA) and GraphPad software (GraphPad Software, Inc., La Jolla, CA, USA) were used to analyse all data for statistical significance. Two‐tailed Student's *t*‐test was used for comparisons of two independent groups. A *P* value <0.05 was considered to indicate statistical significance.

## 3 RESULTS

### 3.1 IRX5 was upregulated in HCC tissues and cell lines

**Figure 1:**
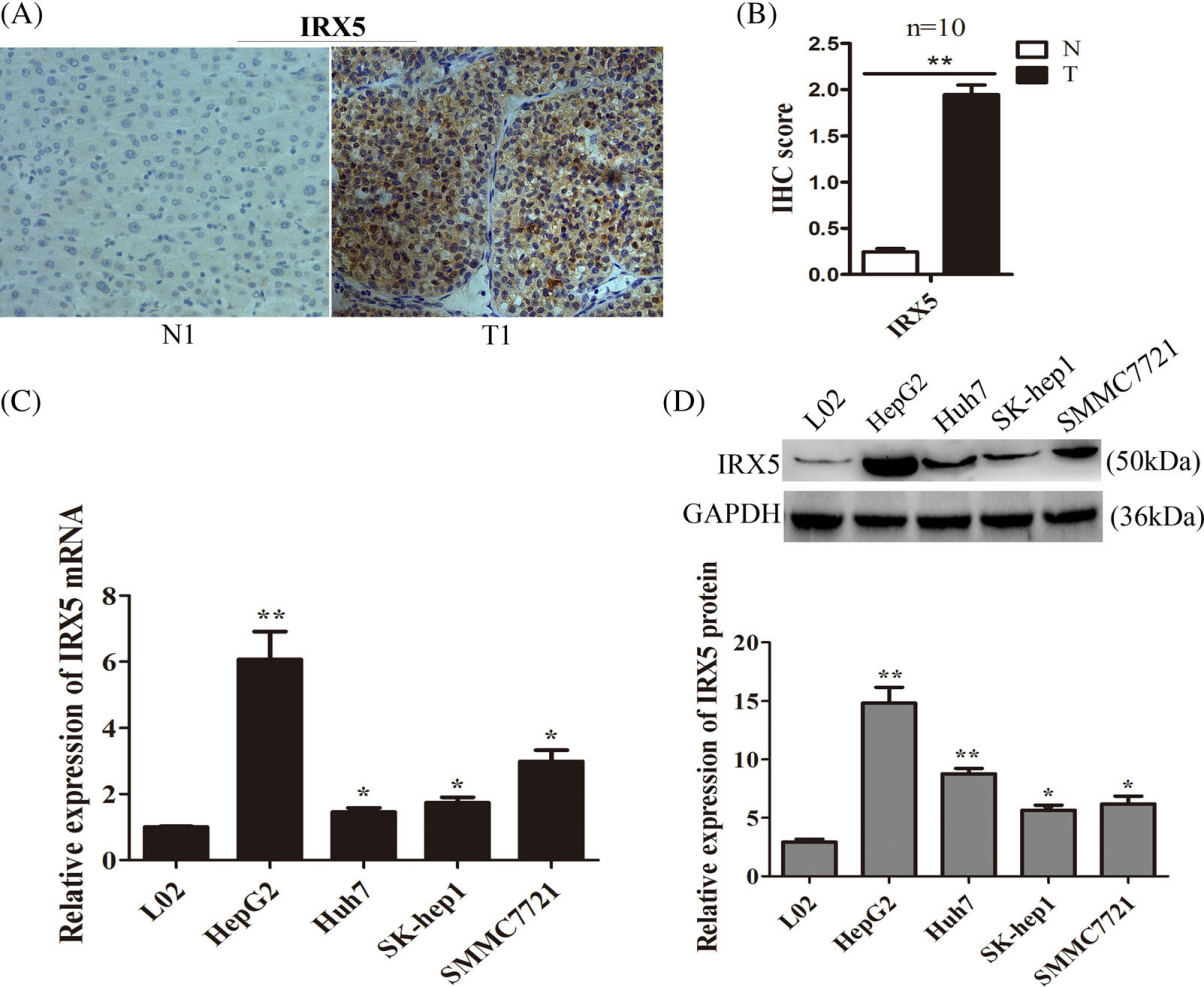
IRX5 was upregulated in HCC tissues and cell lines. (A) Immunohistochemical (IHC) staining of IRX5 in HCC tissues (T) and non‐tumour tissues (N). No. 1 was the representative micrograph. (B) Total IHC score of IRX5 in HCC tissues and non‐tumour tissues (*n* = 10). **P < 0.01. (C) IRX5 mRNA levels were detected by qRT‐ PCR in HCC cell lines(HepG2,huh7, SK‐hep1 and SMMC7721) and human immortalized, normal liver cell line (L02). *P < 0.05, **P < 0.01.Transcript levels were normal to GAPDH expression. (D) IRX5 protein levels in HCC cell lines(HepG2,huh7, SK‐hep1 and SMMC7721) and L02 were detected by western bloting. GAPDH was used as internal control. *P < 0.05, **P < 0.01

Immunohistochemical (IHC) staining revealed that the IRX5 protein was differentially expressed between tumour tissues and their matched adjacent non‐tumour tissues. The non‐tumour tissues showed weak or no expression of IRX5 (Figure 1A, left panel). However, strong immunoreactivity for IRX5 was detected in the cell nucleus and in the cytoplasm of the tumor tissues (Figure 1A, right panel). Total IHC score of IRX5 in HCC tissues and non‐tumour tissues (*n* = 10, *P* < 0.01) (Figure 1B).

QRT‐PCR analysis and western blot analysis were used to investigate theIRX5 expression in HCC cell lines (HepG2, SMMC7721, SK‐hep1, Huh7) and a human immortalized normal liver cell line (L02).The results showed thatIRX5 expression was markably increased in HCC cells compared with that in L02 cells (Figure 1C,D).

### 3.2 IRX5 promoted HCC cell proliferation *in vitro*

**Figure 2:**
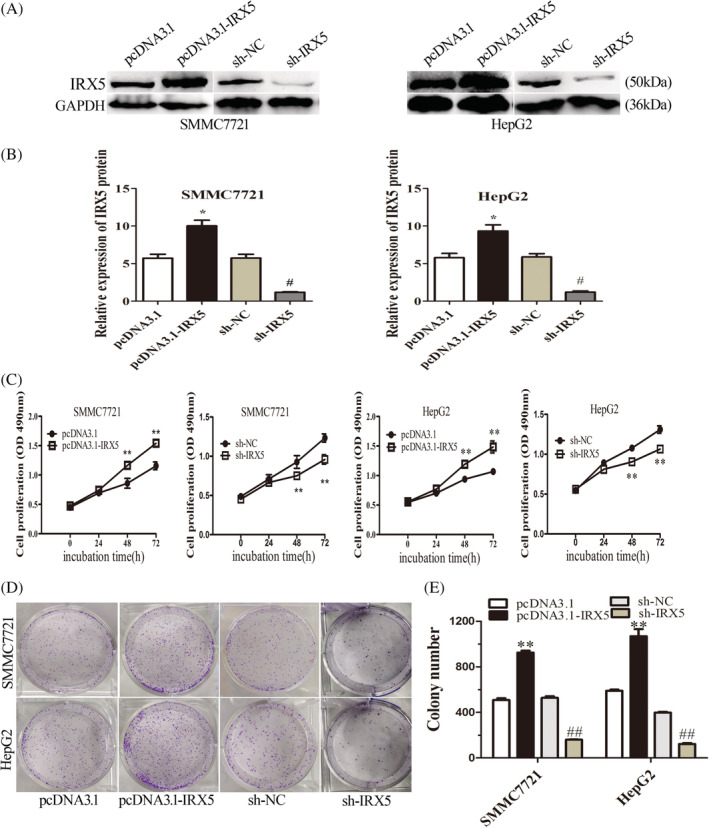
IRX5 promoted HCC cell proliferation *in vitro*. (A,B) Overexpression and knock‐down efficiency of IRX5. GAPDH was used as internal control. (C) Proliferation of HCC cells assessed by MTS assays. IRX5 overexpression enhanced HCC cell proliferation, whereas IRX5 interference repressed HCC cell proliferation. (D,E) Clone formation assay of differently treated HCC cells. Representative graphs are shown. The data graphs depict the count number from three independent experiments

To further understand the correlation between IRX5 expression and the proliferation and apoptosis capacities of HCC cells, we overexpressed IRX5 (pcDNA3.1‐IRX5) and knocked down (sh‐IRX5) via transient transfection in SMMC7721 and HepG2 cells. Western blotting confirmed that transfection with the IRX5 overexpression plasmid significantly increased IRX5 expression (Figure 2A,B). The efficiency of sh‐RNA‐mediated IRX5 knockdown was confirmed (Figure 2A,B).MTS assays showed that the overexpression of IRX5increased the proliferative capacities of SMMC7721 and HepG2 cells compared with control cells containing empty vector, whereas the opposite result was found when IRX5 expression was silenced (Figure 2C). Moreover, IRX5 overexpression enhanced the colony formation capacities of SMMC7721 and HepG2 cells, whereas knockdown of IRX5 reduced them (Figure 2D,E). Together, these data suggest that IRX5 enhanced HCC cell proliferation.

### 3.3 IRX5 induced cell cycle progression by upregulatingcyclinD1 and suppressing apoptosis via inhibiting p53 signalling in HCC cells

**Figure 3:**
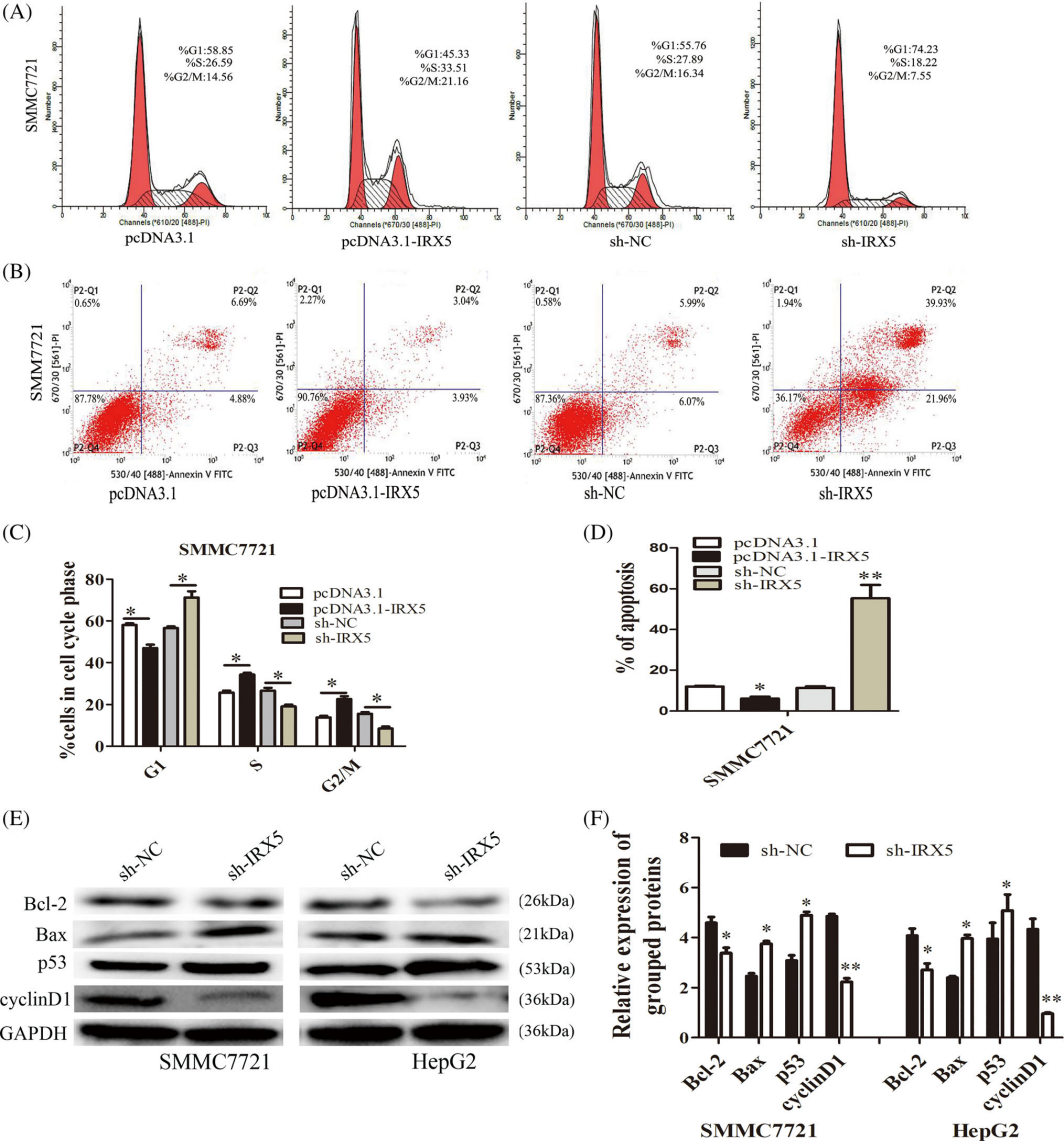
IRX5 induced cell‐cycle progression and suppressed apoptosis. (A,C) Cell‐cycle analysis of differently treated HCC cells. Representative graphs are shown. The data graphs depict the count number from three independent experiments. (B,D) Cell‐apoptosis analysis of differently treated HCC cells. Representative graphs are shown. The data graphs depict the count number from three independent experiments. (E,F) The expression of CyclinD1, P53, Bcl‐2 and Bax in cells were examined by western blotting analyses. GAPDH was used as internal control. *P < 0.05, **P < 0.01

To gain insights into the mechanism by which IRX5 enhances HCC cell proliferation and induces apoptosis, fluorescence‐activated cell sorting (FACS) was performed to analyse differences in cell cycle distributions and apoptosis after IRX5 overexpression or silencing. The results revealed that a reduction in the G1 population and an increase in the S and G2/M populations were observed in SMMC7721 cells overexpressing IRX5. Conversely, repressing IRX5 expression mainly led to an accumulation in G1 and decrease in S and G2/M phases (Figure 3A,C).

Apoptosis was measured by FACS‐based annexin‐V/7‐AAD double staining in HCC cells under serum starvation conditions for 48 hours. The results revealed that the percentage of annexin V‐positive cells was lower in IRX5‐overexpressing SMMC7721 cells than in control cells. In contrast, the sh‐IRX5 group had a significantly higher percentage of annexin V‐positive cells than the sh‐NC group (Figure 3B,D).

Furthermore, we also examined the levels of several key genes involved in cell cycle checkpoints by western blot analysis in SMMC7721 and HepG2 cells stably with silenced IRX5 expression. Knockdown of IRX5 reduced the expression of the oncogenic cell cycle regulator cyclinD1 but increased the expression of the cyclin‐dependent protein kinase inhibitor p53 (Figure 3E,F).

To investigate the role of IRX5 in the p53 signalling pathway, the expression levels of Bax and Bcl‐2 were detected. As shown in Figure 3E,F, increased Bax and decreased Bcl‐2 expressions were observed following downregulation of IRX5 expression. Taken together, these results indicated that IRX5‐induced cell cycle progression by upregulating cyclinD1 and inhibited apoptosis by inactivating the p53 signalling pathway in HCC cells.

### 3.4 Knockdown of IRX5 suppressed HCC tumour growth *in vivo*

**Figure 4:**
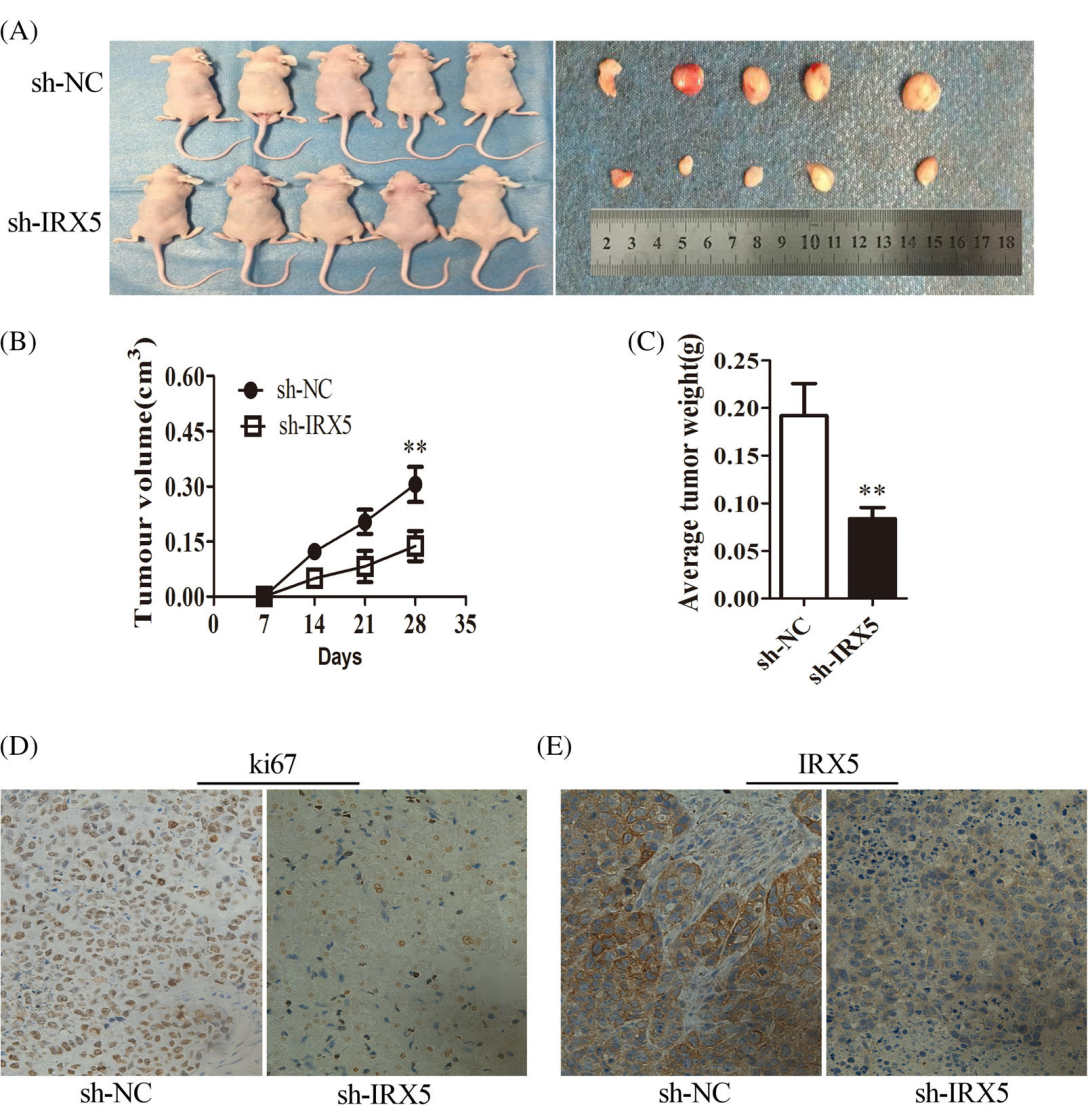
IRX5 silence inhibited HCC growth *in vivo*. (A) The subcutaneous tumour model of stable IRX5‐interference SMMC7721 cells (*n* = 5 for both groups). (B,C) Tumour growth and tumour weights curves were analysed. (D,E) Immunohistochemistry analysis of ki‐67and IRX5 were obtained from tumours (magnification, 400×; scale bar, 50 μm)

To determine the growth‐enhancing effect of IRX5 *in vivo*, we injected SMMC7721 cells stably transfected with sh‐IRX5 or sh‐NC subcutaneously into nude mice for xenotransplantation. Mice injected with IRX5‐silenced cells showed significantly decreased tumour growth compared to those injected with cells transfected with sh‐NC (Figure 4A). As assessed by measurements of tumour volume and weight (Figure 4B,C), the knocked down of IRX5 expression significantly inhibited overall tumour growth. The immunohistochemistry analysis of the tumour tissues from xenografts revealed that the expression of the cell proliferation marker, Ki‐67, was significantly weaker in xenografts of sh‐IRX5 cells than in xenografts of sh‐NC cells (Figure 4D,E). These results showed that knockdown of IRX5 suppressed HCC cell proliferation *in vivo*.

## 4 DISCUSSION

Our study demonstrates that IRX5 is upregulated in HCC cell lines and HCC tissues. Furthermore, IRX5 promoted cell proliferation *in vitro* and *in vivo*. In addition, IRX5 suppressed apoptosis *in vitro*. IRX5 plays a different role in multiple cancers, contributing to the development of many tumours by acting as an important transcription factor regulating key regulatory genes that control cell growth, invasion, migration and apoptosis. IRX5 activity is also present in the stromal and proliferative late blastemal/early epithelial cells in developing kidneys and Wilms tumours.^16^ Myrthue et al had revealed that knockdown of IRX5 by RNA interference significantly reduction in LNCaP cell viability, which resulted in increased LNCaP cell apoptosis and was partially mediated by p53.^17^ However, the role of IRX5 in HCC is unknown. In this study, we provided the first evidence that IRX5 is significantly upregulated in HCC cell lines and tissues. We further identified the effects of IRX5 on the biological behaviours of HCC cells, showing that IRX5 promoted HCC cell proliferation and inhibited apoptosis. The results indicated that IRX5 acts as an oncogene in HCC. Moreover, the *in vivo* studies also confirmed that knockdown of IRX5 suppressed tumour growth in nude mice, suggesting that IRX5 could potentially be applied in the treatment of HCC. However, the underlying mechanisms by which IRX5 promotes tumour cell proliferation and inhibits apoptosis remain unclear.

We provided evidence that IRX5 promoted HCC cell proliferation *in vitro* and *in vivo*. Furthermore, our study found that a reduction in the G1 population and an increase in the S and G2/M populations were observed in SMMC7721 cells overexpressing IRX5. Mechanistically, IRX5 was shown to induce cell cycle progression by upregulatingcyclinD1. It has been well defined that tumour‐associated cell cycle defects are often mediated by the accumulation of cyclins (CCNs).^18^ Cyclins are divided into two groups known as the G1/S cyclins, which are essential for the control of the cell cycle at the G1 to S transition, and the G2/M cyclins, which control the cell cycle at the G2 to M transition phase.^19^ Cyclin D1 is a major regulator of the cell cycle and is responsible for the G1/S‐phase transition.^20^ Ao et al reported that cyclin D1 was overexpressed in liver cancer cells and promoted migration and invasion by regulating several enzymes.^21^ Chen et al also reported that HCC tissues and HCC cells exhibited elevated expression levels of cyclin D1 and its expression levels were found to be correlated with tumour size and tumour staging.^22^ A previous study reported that IRX5 promotes NSCLC cell proliferation by means of regulating the CCND1 promoter.^9^ In this study, cyclin D1 expression was positively correlated with the expression of IRX5 in HCC cells.

In addition to enhanced proliferation, resistance to apoptosis is also a hallmark of cancer cells.^23^ The p53 gene is located on human chromosome 17p13 and is a tumour suppressor and pro‐apoptosis gene. Wild‐type p53 plays a key role in cell gene transcription, cell cycle regulation, apoptosis, proliferation and differentiation.^24^, ^25^, ^26^ The amount of wild‐type p53 protein is low in normal cells.^27^ The dominant components of p53 signalling, including p53, Bcl‐2 and Bax, have been extensively studied in carcinomas.^28^, ^29^ A previous study reported that p53 positively regulates Bax expression^30^ and negatively regulates the transcription of Bcl‐2.^15^, ^30^ Thus, p53 is likely to affect upstream pro‐apoptotic proteins to modulate their functions in the cytoplasm. We also found that the downregulation of IRX5 significantly increased the expression of p53 and Bax and decreased the expression of Bcl‐2 in HCC cells. However, in our study, we did not study the mechanism by whichIRX5 inhibits the p53 signalling pathway. Such mechanisms are left to be investigated in future studies.

In summary, our study demonstrated that IRX5 is a potential tumour promoter gene in HCC. IRX5 promotes to the promoter of carcinogenesis by facilitating cell proliferation and suppressing cell apoptosis by inhibiting the p53 signalling pathway. As a result, IRX5 might act as a novel molecular target for the detection and treatment of HCC. However, further studies are needed to investigate the effect of IRX5 on other cellular processes in HCC, such as cell adhesion and differentiation.

## 5 CONCLUSION

Our results showed that IRX5 was upregulated in HCC and cells. Overexpression of IRX5 promoted cell proliferation and tumourigenicity. Knockdown of IRX5 promoted cell apoptosis through the p53 signalling pathway in HCC.

## CONFLICT OF INTEREST

The authors have declared that no competing interests exist.

6

## Data Availability

All data and models generated or used during the study appear in the submitted article.

## References

[1] Bray F , Ferlay J , Soerjomataram I , Siegel RL , Torre LA , Jemal A . Global cancer statistics 2018: GLOBOCAN estimates of incidence and mortality worldwide for 36 cancers in 185 countries. CA Cancer J Clin. 2018;68(6):394‐424. 30207593 10.3322/caac.21492

[2] Chen LC , Chiou WY , Lin HY , et al. Comparing stereotactic ablative radiotherapy (SABR) versus re‐trans‐catheter arterial chemoembolization (re‐TACE) for hepatocellular carcinoma patients who had incomplete response after initial TACE (TASABR): a randomized controlled trial. BMC Cancer. 2019;19(1):275. 30922261 10.1186/s12885-019-5461-3 PMC6437913

[3] Zhu L , Liu Y , Chen Q , et al. Long‐noncoding RNA colorectal neoplasia differentially expressed gene as a potential target to upregulate the expression of IRX5 by miR‐136‐5P to promote oncogenic properties in hepatocellular carcinoma. Cell Physiol Biochem. 2018;50(6):2229‐2248. 30423553 10.1159/000495084

[4] Xu G , Zhu L , Wang Y , Shi Y , Gong A , Wu C . Stattic enhances radiosensitivity and reduces radio‐induced migration and invasion in HCC cell lines through an apoptosis pathway. Biomed Res Int. 2017;2017:1832494. 29226125 10.1155/2017/1832494 PMC5684518

[5] Dang S , Zhou J , Chen Y , et al. Dynamic expression of ZNF382 and its tumor‐suppressor role in hepatitis B virus‐related hepatocellular carcinogenesis. Oncogene. 2019;38(24):4804‐4819. 30804458 10.1038/s41388-019-0759-9

[6] Hu X , Tang Z , Ma S , Yu Y , Chen X , Zang G . Tripartite motif‐containing protein 7 regulates hepatocellular carcinoma cell proliferation via the DUSP6/p38 pathway. Biochem Biophys Res Commun. 2019;511(4):889‐895. 30850165 10.1016/j.bbrc.2019.02.001

[7] Yang L , Qiu J , Xiao Y , et al. AP‐2β inhibits hepatocellular carcinoma invasion and metastasis through slug and snail to suppress epithelial‐mesenchymal transition. Theranostics. 2018;8(13):3707‐3721. 30026878 10.7150/thno.25166 PMC6037033

[8] Huang L , Song F , Sun H , Zhang L , Huang C . IRX5 promotes NF‐κB signalling to increase proliferation, migration and invasion via OPN in tongue squamous cell carcinoma. J Cell Mol Med. 2018;22(8):3899‐3910. 10.1111/jcmm.13664 PMC6050492 29761910

[9] Zhang DL , Qu LW , Ma L , et al. Genome‐wide identification of transcription factors that are critical to non‐small cell lung cancer. Cancer Lett. 2018;434:132‐143. 30031117 10.1016/j.canlet.2018.07.020

[10] Ahmadian N , Pashaei‐Asl R , Samadi N , et al. Hesa‐a effects on cell cycle signaling in esophageal carcinoma cell line. Middle East J Dig Dis. 2016;8(4):297‐302. 27957293 10.15171/mejdd.2016.39 PMC5145297

[11] Diehl JA . Cycling to cancer with cyclin D1. Cancer Biol Ther. 2002;1(3):226‐231. 12432268 10.4161/cbt.72

[12] Klein EA , Assoian RK . Transcriptional regulation of the cyclin D1 gene at a glance. J Cell Sci. 2008;121(Pt 23):3853‐3857. 19020303 10.1242/jcs.039131 PMC4545630

[13] Koseoglu RD , Sezer E , Eyibilen A , Aladag I , Etikan I . Expressions of p53, cyclinD1 and histopathological features in basal cell carcinomas. J Cutan Pathol. 2009;36(9):958‐965. 19187116 10.1111/j.1600-0560.2008.01204.x

[14] Yudhani RD , Astuti I , Mustofa M , Indarto D , Muthmainah M . Metformin modulates cyclin D1 and P53 expression to inhibit cell proliferation and to induce apoptosis in cervical cancer cell lines. Asian Pac J Cancer Prev. 2019;20(6):1667‐1673. 31244286 10.31557/APJCP.2019.20.6.1667 PMC7021606

[15] Akhtar RS , Geng Y , Klocke BJ , et al. BH3‐only proapoptotic Bcl‐2 family members Noxa and Puma mediate neural precursor cell death. J Neurosci. 2006;26(27):7257‐7264. 16822983 10.1523/JNEUROSCI.0196-06.2006 PMC6673947

[16] Holmquist Mengelbier L , Lindell‐Munther S , Yasui H , et al. The Iroquois homeobox proteins IRX3 and IRX5 have distinct roles in Wilms tumour development and human nephrogenesis. J Pathol. 2019;247(1):86‐98. 30246301 10.1002/path.5171 PMC6588170

[17] Myrthue A , Rademacher BL , Pittsenbarger J , et al. The Iroquois homeobox gene 5 is regulated by 1,25‐dihydroxyvitamin D3 in human prostate cancer and regulates apoptosis and the cell cycle in LNCaP prostate cancer cells. Clin Cancer Res. 2008;14(11):3562‐3570. 18519790 10.1158/1078-0432.CCR-07-4649

[18] Bonelli P , Tuccillo FM , Borrelli A , Schiattarella A , Buonaguro FM . CDK/CCN and CDKI alterations for cancer prognosis and therapeutic predictivity. Biomed Res Int. 2014;2014:361020. 24605326 10.1155/2014/361020 PMC3925518

[19] Galderisi U , Jori FP , Giordano A . Cell cycle regulation and neural differentiation. Oncogene. 2003;22(33):5208‐5219. 12910258 10.1038/sj.onc.1206558

[20] Lian J , Tian H , Liu L , et al. Downregulation of microRNA‐383 is associated with male infertility and promotes testicular embryonal carcinoma cell proliferation by targeting IRF1. Cell Death Dis. 2010;1:e94. 21368870 10.1038/cddis.2010.70 PMC3032325

[21] Ao R , Zhang DR , Du YQ , Wang Y . Expression and significance of Pin1, beta‐catenin and cyclin D1 in hepatocellular carcinoma. Mol Med Rep. 2014;10(4):1893‐1898. 25109821 10.3892/mmr.2014.2456

[22] Chen J , Li X , Cheng Q , et al. Effects of cyclin D1 gene silencing on cell proliferation, cell cycle, and apoptosis of hepatocellular carcinoma cells. J Cell Biochem. 2018;119(2):2368‐2380. 28885717 10.1002/jcb.26400

[23] Evan GI , Vousden KH . Proliferation, cell cycle and apoptosis in cancer. Nature. 2001;411(6835):342‐348. 11357141 10.1038/35077213

[24] Shangary S , Wang S . Targeting the MDM2‐p53 interaction for cancer therapy. Clin Cancer Res. 2008;14(17):5318‐5324. 18765522 10.1158/1078-0432.CCR-07-5136 PMC2676446

[25] Nag S , Zhang X , Srivenugopal KS , Wang MH , Wang W , Zhang R . Targeting MDM2‐p53 interaction for cancer therapy: are we there yet? Curr Med Chem. 2014;21(5):553‐574. 24180275 10.2174/09298673113206660325 PMC6690199

[26] Shangary S , Qin D , McEachern D , et al. Temporal activation of p53 by a specific MDM2 inhibitor is selectively toxic to tumors and leads to complete tumor growth inhibition. Proc Natl Acad Sci USA. 2008;105(10):3933‐3938. 18316739 10.1073/pnas.0708917105 PMC2268798

[27] Li Z , Han C , Feng J . Relationship of the expression levels of XIAP and p53 genes in hepatocellular carcinoma and the prognosis of patients. Oncol Lett. 2017;14(4):4037‐4042. 28959363 10.3892/ol.2017.6681 PMC5607648

[28] Jin Y , Cao B , Zhang M , et al. RASSF10 suppresses hepatocellular carcinoma growth by activating P53 signaling and methylation of RASSF10 is a docetaxel resistant marker. Genes Cancer. 2015;6(5–6):231‐240. 26124922 10.18632/genesandcancer.67 PMC4482244

[29] Ou X , Lu Y , Liao L , et al. Nitidine chloride induces apoptosis in human hepatocellular carcinoma cells through a pathway involving p53, p21, Bax and Bcl‐2. Oncol Rep. 2015;33(3):1264‐1274. 25530218 10.3892/or.2014.3688

[30] Mirzayans R , Andrais B , Scott A , Murray D . New insights into p53 signaling and cancer cell response to DNA damage: implications for cancer therapy. J Biomed Biotechnol. 2012;2012:170325. 22911014 10.1155/2012/170325 PMC3403320

